# The development of a machine learning algorithm to identify occupational injuries in agriculture using pre-hospital care reports

**DOI:** 10.1007/s13755-021-00161-9

**Published:** 2021-07-29

**Authors:** Erika Scott, Liane Hirabayashi, Alex Levenstein, Nicole Krupa, Paul Jenkins

**Affiliations:** 1 Northeast Center for Occupational Health and Safety in Agriculture, Forestry, and Fishing, Bassett Medical Center, Cooperstown, NY USA; 2 Bassett Research Institute, Bassett Medical Center, Cooperstown, NY USA; 3 Consultant, Denver, CO USA

**Keywords:** Occupational epidemiology, Injury surveillance, Agriculture, Machine learning

## Abstract

**Purpose:**

Current injury surveillance efforts in agriculture are considerably hampered by the limited quantity of occupation or industry data in current health records. This has impeded efforts to develop more accurate injury burden estimates and has negatively impacted the prioritization of workplace health and safety in state and federal public health efforts. This paper describes the development of a Naïve Bayes machine learning algorithm to identify occupational injuries in agriculture using existing administrative data, specifically in pre-hospital care reports (PCR).

**Methods:**

A Naïve Bayes machine learning algorithm was trained on PCR datasets from 2008–2010 from Maine and New Hampshire and tested on newer data from those states between 2011 and 2016. Further analyses were devoted to establishing the generalizability of the model across various states and various years. Dual visual inspection was used to verify the records subset by the algorithm.

**Results:**

The Naïve Bayes machine learning algorithm reduced the volume of cases that required visual inspection by 69.5 percent over a keyword search strategy alone. Coders identified 341 true agricultural injury records (Case class = 1) (Maine 2011–2016, New Hampshire 2011–2015). In addition, there were 581 (Case class = 2 or 3) that were suspected to be agricultural acute/traumatic events, but lacked the necessary detail to make a certain distinction.

**Conclusions:**

The application of the trained algorithm on newer data reduced the volume of records requiring visual inspection by two thirds over the previous keyword search strategy, making it a sustainable and cost-effective way to understand injury trends in agriculture.

## Introduction

Quantifying occupational injuries are of particular importance in the agricultural sector, which has one of the highest fatality rates in the United States [1]. While workplace fatalities are often captured in the Census of Fatal Occupational Injury (CFOI) [2], collecting data relating to injury, especially in Northeast agriculture, is problematic [3]. The majority of farms in the Northeast are exempt from OSHA regulations, as only 10.7% of Northeast region farms with hired workers have 10 or more employees [4]. The injury and fatality estimates that do exist are limited [5–8]. Similarly, data has also shown underreporting of injuries in the forestry and logging sector, which is part of the agricultural super-sector [9].

Surveillance efforts have been considerably constrained by a lack of knowledge regarding what information to collect and where to find it. These gaps in knowledge have impeded efforts to develop more accurate injury burden estimates and have negatively impacted the prioritization of workplace health and safety in state and federal public health efforts [10]. The low prioritization of occupational health and safety is demonstrated by NIOSH’s 0.2% share of all medical research and development expenditures in the 2016 federal budget [11, 12]. By creating a system to identify previously unreported agriculture related injury, it will be possible to provide a more complete picture of injury burden in this high-risk industry.

The loss of several national surveillance efforts—the National Agricultural Worker Health Survey Injury Module [13], and the Occupational Injury Surveillance of Production Agriculture (OISPA) Survey [14]—further justifies the need to invest in new strategies for surveillance research. With robust computing power now available for little cost and the ability to obtain existing administrative health databases with free-text, artificial intelligence and machine learning methods offer promising avenues for public health research. For example, Koivu and Sairanen’s use of several machine learning methods to develop a model that predicts early still birth and pre-term pregnancies [15]. Rybinski et al. applied natural language processing methods to identifying family members and diseases in free text family history sections of electronic health records [16]. Prieto et al. developed a natural language processing method using logistic regression and several keywords to identify opioid misuse in the narrative portion of PCRs [17]. Yang et al. developed a deep neural network to identify cases involving allergic reactions in the free text section of hospital safety reports [18]. This paper describes the development of a Naïve Bayes machine learning algorithm for pre-hospital care reports (PCR) to identify agricultural occupational injuries along with the algorithm’s utility on untagged datasets. Naïve Bayes methodology was chosen for our first effort utilizing machine learning because of its simplicity and effectiveness in text classification [19, 20].

## Materials and methods

The creation of a”gold-standard’ dataset has been described in detail elsewhere and will be briefly summarized here [21]. Over 50,000 PCR records were visually inspected and tagged as to their occupational injury status. In order to be included in this review, the record’s narrative needed to contain a keyword from Table 1. This tagged dataset, which came from Maine and New Hampshire 2008–2010 PCRs, served as the basis for training the machine learning algorithm. The following describes the training of the algorithm on this tagged dataset, and the subsequent application of this trained algorithm to newer datasets for which case determination had not yet been made. These newer datasets included Maine PCR data from 2011–2016 and New Hampshire PCR data from 2011–2015. The Institutional Review Board (IRB) of the Mary Imogene Bassett Hospital approved all protocols. Additionally, approval was also granted by each participating state’s IRB or data use board.

**Table 1:** Stemmed keywords

3_Point_hitch	Chain	Farmer	Hors	Plowshar	Stall
Agricultur	Chain_saw	Feed	Implement	Poultri	Straw
Anim	Chainsaw	Fenc	Irrig	Prune	Tedder
Arch	Chicken	Fenc_post	Kickback	Pto	Three_point_hitch
Auger	Choker	Fertil	Kicker	Ram	Tie_down
Bale	Chute	Fop	Limb	Sanit	Timber
Barn	Cleanser	Forestri	Livestock	Scraper	Tractor
Beater	Combin	Gator	Loader	Shear	Tree
Bind	Compost	Gear	Log	Sheav	Trough
Blade	Corral	Goat	Logger	Sheep	Turkey
Bobcat	Coveral	Grain_bin	Manur	Silag	Udder
Breed	Cow	Greenhous	Methan	Silo	Uncap
Buck	Crop	Guywir	Milk	Skid_steer	Unhitch
Buggi	Dairi	Harrow	Mower	Skidder	Vacuum_pump
Bull	Debark	Hay	Pastur	Skidsteer	Wagon
Bulldoz	Defac	Hitch	Pen	Slaughter	Winch
Bunker	Digger	Hog	Pesticid	Splitter	Wood
Cabl	Drive_line	Hoof	Pig	Sprayer	Yard
Calv	Entangl	Hoof_trimmer	Pipelin	Spreader	Yearl
Cart	Farm	Hoov	Plow	Spring_pole	

### Preprocessing of all datasets used in these analyses

Cleaning PCR records involved two steps: (1) removing duplicates and (2) removing records of no interest. Duplicate records were identified based on an exact match on four variables: gender, admission, ZIP code, and date of birth. For records that met these criteria, one was retained at random. PCR records of no interest included those with a dispatch reason of transfer, lifting, or intercept; or those with destination type of nursing home.

### Training the algorithm using tagged datasets

The algorithm was trained on the gold standard PCR dataset from 2008 to 2010 from Maine and New Hampshire using the variables shown in Table 2. After PCR records were cleaned using SAS 9.3 (Cary, NC), they were imported into Python (v 3.7) for further data processing. Three Python packages were used: pandas (v 1.1.1) for data management, nltk (v 3.5) for natural language processing, and scikit-learn (v 0.23.2) for machine learning.

**Table 2:** Variables within the dataset

Incident location
Mechanism of injury
Dispatch reason
Primary impression
Stemmed Keywords (Table 1)
Gender
Admit date
Date of birth
Zip code
State

Transformation mapping was performed to link similar variables (Table 2) across state datasets between given years of data. These maps were applied to the following variables: incident location, mechanism of injury, dispatch reason, and primary impression. For the other variables in Table 2, a dummy matrix was created which included a (0,1) variable for each level of the variable they represented, a process known as one-hot encoding. For example, the fifty-nine levels for mechanism of injury were represented by fifty-nine (0,1) variables.

Narratives were prepared for the stemmed keyword search by lowercasing all characters, removing all punctuation, and stemming all words using the Natural Language Toolkit’s (NLTK) Snowball stemmer [22]. Next, narratives were scanned for any instances of the stemmed keywords in Table 1. Throughout this process, the algorithm was trained to ignore keywords that were found in combination with other words or phrases that did not indicate an agricultural injury, such as proper names of emergency responders, local non-agricultural businesses, or keywords followed by a known address suffix or abbreviation [23]. Lastly, exclusions were applied for irrelevant words that stemmed to the identical value as a given keyword (e.g., “animate” and “animal” both stem to “anima”; therefore “animate” was excluded). Based on that searching process, each narrative was tagged as to the presence (1) or the absence (0) of each of the stemmed keywords in Table 1.

A four-level case-class variable was created as follows: 0 (non-agricultural, non-traumatic/acute, or both), 1 (confirmed agricultural, confirmed traumatic/acute = true case), 2 (confirmed traumatic/acute, suspected agricultural), or 3 (suspected traumatic/acute, confirmed agricultural) (Table 3). Naïve Bayes models were run for binary case (case-class 1,2, or 3 versus case-class 0). These models used all of these variables in conjunction to assign a predicted probability that the record was a true case.

**Table 3:** Case class choices

Case determination	Description
0—not a case	0 (non-agricultural, non-traumatic/acute, or both)
1—Agriculture	1 (confirmed agricultural, confirmed traumatic/acute = true case)
2—Agriculture	2 (confirmed traumatic/acute, suspected agricultural)
3—Agriculture	3 (suspected traumatic/acute, confirmed agricultural)

The essential element of these analyses was to identify variables that occurred relatively frequently in cases and relatively infrequently in non-cases. Using this mechanism, our goal was to train the algorithm to identify a posterior probability threshold for assigning a record to be a true case such that ninety percent (90%) of all true cases would be identified. To determine what threshold would meet this ninety percent (90%) requirement, the algorithm was trained on eighty percent (80%) of the data selected at random, and validated on the remaining twenty percent (20%). This procedure was repeated for one hundred iterations. Over these one hundred iterations, the mean and standard deviation were calculated for the required threshold probability [in our case this is 0.17, discussed further in the results section]. The mean and standard deviation of the percentage of all cases in the dataset meeting this threshold probability were also calculated. Hypothetically, on iteration three, in order for the algorithm to identify a subset of the records that contained ninety percent (90%) of the true positive cases it was necessary to “tag” any record with a posterior probability of 0.17 or higher as a “hit”. This resulted in three percent (3%) of all records in this iteration being tagged as “hits”. Therefore, the relevant data points for this iteration were 0.17 (the required threshold posterior probability), and 0.03 (the proportion of all records that the algorithm needed to assign as “hits” in order to capture 90% of the true hits in the file).

The proportion of records within the file that was tagged by the algorithm as “hits” that had been previously confirmed as case-class 1, 2, or 3 was also recorded. As a hypothetical example, on iteration three, the algorithm was training on a file containing one hundred (100) records that were confirmed case-class 1, 2, or 3. In order for the algorithm to identify a subset containing ninety (90) of these confirmed case-class 1, 2, or 3 records, 3,012 records with a posterior probability of 0.17 or greater were tagged as “hits”. The resulting percentage was therefore 90/3,012 = 0.0299 or 3%.

A receiver operator characteristic curve (ROC) with sensitivity (true positive rate) on the y-axis and 1-specificity (false positive rate) on the x-axis was also created for certain of these iterations. The area under these curves (AUC) was also recorded as an additional data point.

Sub-analyses were also performed with the goal of identifying the relative importance (discriminatory power) of the variables in Table 2. Further analyses were devoted to establishing the generalizability of the model across various states and various years. Variable importance was determined by subtracting the log probability of the negative class for each variable from the log probability of the positive class. This gave a measure of how strong a discriminator the variable was.

### Application of the trained algorithm on newer un-tagged datasets from Maine (2011–2016) and New Hampshire (2011–2015)

For each of the new untagged datasets (PCR data from Maine 2011–2016 and New Hampshire 2011–2015), the trained algorithm was used to identify the subset of records that had a posterior probability that was equal to or greater than the mean of the one-hundred (100) threshold probabilities obtained above. These records were set aside for visual inspection to determine their true case classification as being either a 1, 2, or 3 versus 0 (Table 3).

### Visual confirmation of records meeting the probability threshold as identified by the trained algorithm

The visual case determination utilized the following variables: state ID, state, incident ID, date of birth, gender, incident location, dispatch reason, primary impression, mechanism of injury, incident date (admit date), stems, narrative. The research team developed an injury surveillance manual with specific coding rules, and this manual was updated throughout the visual case determination process to address any questions that arose. Trained coders independently assigned one of the four case-class levels (Table 3) to each record.

Any case that was not assigned a zero (not a case of interest) received a second, separate review by an additional coder; and discrepancies between the two case determinations were resolved by the two coders. When the initial review was complete, all non-zero cases and a random sample of 10% of zero cases were reviewed again by lead reviewers (Principal Investigator and Research Coordinator) to confirm the case determination. The lead review results were also used to provide additional training to the coders and to update the injury surveillance manual. In addition, coders used comment fields to suggest new exclusions; and these exclusion suggestions were reported to the study team, and subsequently included in future iterations of the algorithm’s exclusion list. The labor resource allocation to visual case determination was calculated in minutes per case, then transformed into full-time equivalents (FTE). For each of the untagged datasets, the percent of records in these tagged files that were confirmed by visual examination to be case-class 1, 2, or 3 was recorded.

## Results

### Results from the training of the algorithm on data from 2008 to 2010

Of the total of 1,072,745 records, 224,572 (20.9%) were eliminated in SAS as being either duplicates or irrelevant, leaving 848,173 records for the application of the algorithm. As the algorithm was trained, a total of 1557 exclusion word or phrases were identified.

The average posterior probability cutoff over the training iterations that was required to produce a sub-dataset that contained ninety percent of all true positives was 0.016 (SD 0.007). On average, fifteen percent (15%) of records fed into the model had a posterior probability > 0.016 and were thus reviewed visually. Averaged over the 100 iterations, 10.6% (SD 2.64) of the records with a posterior probability of 0.016 or higher were found to be agricultural cases.

**Fig. 1:**
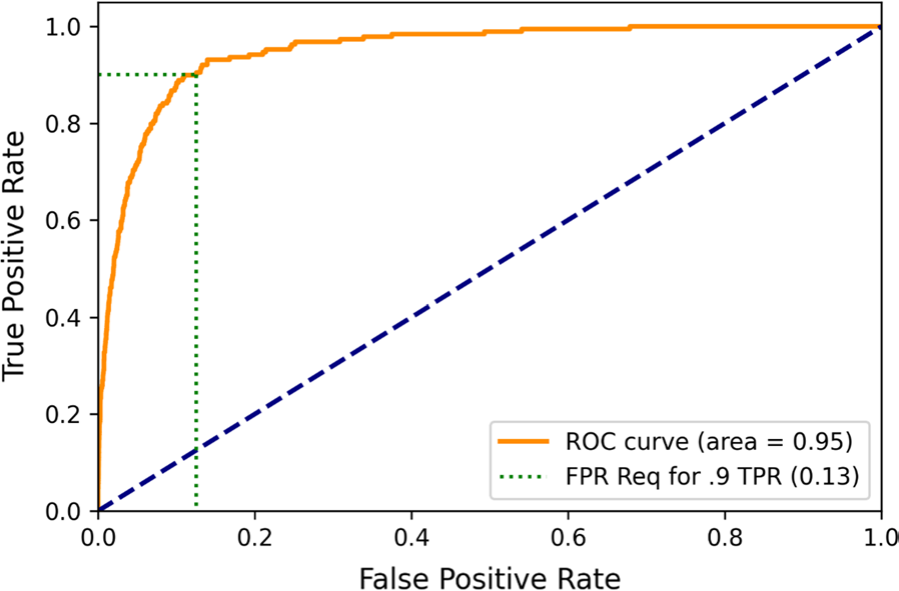
Receiver Operator Characteristic Curve for Naïve Bayes Tested on 2008 and 2010 Data from Maine & New Hampshire, Trained on 2009

**Fig. 2:**
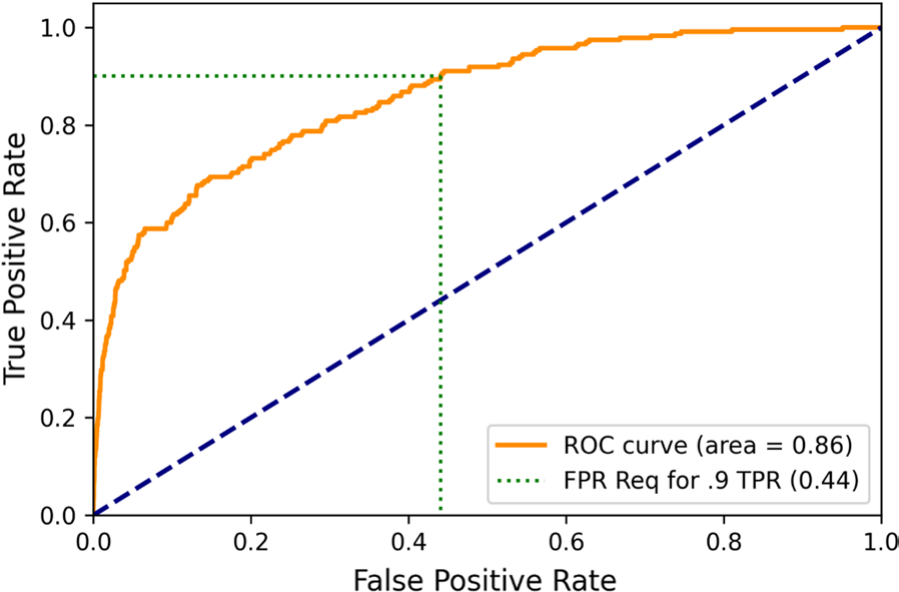
Receiver Operator Characteristic Curve for Naïve Bayes Tested on New Hampshire (2008–2010), Trained on Maine

The mean and standard deviation of the area under the ROC over the 100 80%—20% training-validation iterations were mean 0.95 and SD 0.01 respectively. The corresponding AUC values for the across year (train 2008, 2010 test on 2009) and across state (trained on Maine, tested on New Hampshire) iterations were 0.94 and 0.85 respectively (Figs. 1 and 2, Table 4).

**Table 4:** Results from employing various variables in the naïve bayes machine learning model

Train scenario	Test scenario	Required true positive rate	Necessary false positive rate	AUC
2008, 2009	2010	0.9	0.17	0.93
2008, 2010	2009	0.9	0.13	0.95
2009, 2010	2008	0.9	0.21	0.94
Maine 2008–2010	New Hampshire 2008–2010	0.9	0.44	0.86
New Hampshire 2008–2010	Maine 2008–2010	0.9	0.45	0.83

### Results from application of the trained algorithm on the newer un-tagged datasets from 2011 to 2016

**Fig. 3:**
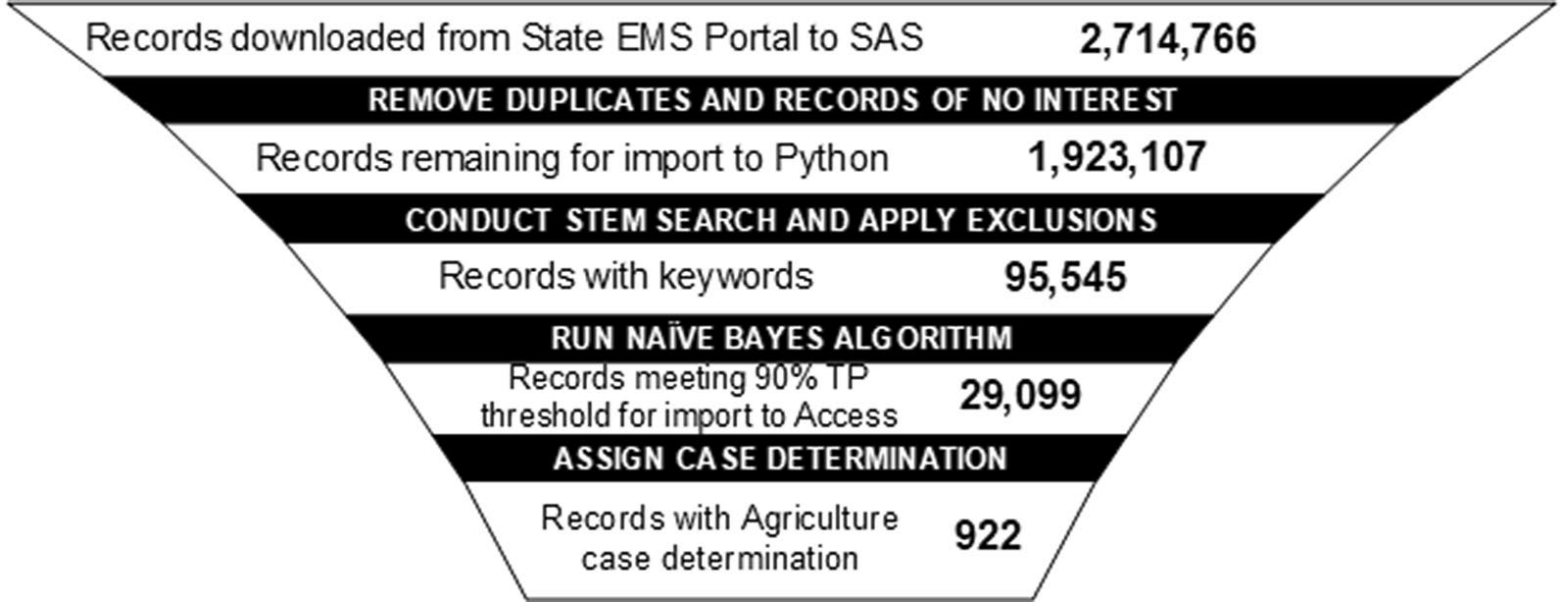
Reduction of 2011–2015(6) Data Through NEC Surveillance System

Eliminating duplicates and records of no interest reduced the volume of records for machine learning by 791,659 (Fig. 3). A total of 1,923,107 PCR records were imported into Python. Of these records, 95,545 contained a stemmed keyword of interest. The Naïve Bayes algorithm identified 29,099 records (30.5%) that had a posterior probability of being an agricultural case of 0.016 or greater and therefore met the 90% true positive threshold that was derived from the training of the algorithm on the 2008–2010 data.

#### Results from visual confirmation of records

Visual inspection of these 29,099 records subsequently confirmed that 922 (3.2%) were in fact agricultural cases. The time burden for case determination is shown in Table 5. Initial case determination of the 29,099 records requiring visual inspection was estimated to require 1.7 full-time equivalent (FTE) staff. The FTE required if we did not employ machine learning—visually inspecting 95,545 records–would have been 5.6 FTE. There is a small additional amount of time for discrepancy resolution and lead review inspection, but that is a fraction of the time required for initial case determination.

**Table 5:** Burden for case determination (per record)

Role	Activity	Average Time (minutes/record)
Initial reviewer	First and second coding	3.5
	Discrepancy review	6
Lead reviewer	Verifying initial case determination	2.5
	Reviewing case determination questions	5

Of the 922 records confirmed to be agricultural cases, coders identified 341 true agricultural injury records (Case class = 1) (Maine 2011–2016, New Hampshire 2011–2015). In addition, there were 581 (Case class = 2 or 3) that were suspected to be agricultural acute/traumatic events, but lacked the necessary detail to make a certain distinction. The top twenty variables with the highest discriminatory power of a true agricultural case are summarized in Table 6.

**Table 6:** Top twenty variables in terms of discriminatory power (training dataset)

Variable	Log Probability Difference	Variable Positive/Target Positive	Variable Positive/Target Negative	Variable Negative/Target Positive	Variable Negative/Target Negative
Stem: hoov	5.284761681	3	0	529	31,303
Stem: silag	4.5916145	1	0	531	31,303
Stem: three_point_hitch	4.5916145	1	0	531	31,303
Stem: grain_bin	4.5916145	1	0	531	31,303
Stem: hoof_trimmer	4.5916145	1	0	531	31,303
Stem: plowshar	4.5916145	1	0	531	31,303
Stem: 3_point_hitch	3.89846732	1	1	531	31,302
Stem: cow	3.62817699	28	37	504	31,266
Stem: slaughter	3.610785247	2	3	530	31,300
Stem: choker	3.493002212	1	2	531	31,301
Stem: harrow	3.493002212	1	2	531	31,301
Primaryimpression_Cardiac—Ventricular Fibrillation	3.493002212	1	2	531	31,301
Primaryimpression_Traumatic Injury—Electrocution	3.493002212	1	2	531	31,301
Incidentloc_Farm	3.352703645	134	232	398	31,071
Stem: hay	3.258429964	28	54	504	31,249
Stem: pastur	3.205320139	8	17	524	31,286
Stem: skid_steer	3.205320139	2	5	530	31,298
Stem: udder	3.205320139	0	1	532	31,302
Primaryimpression_Traumatic Injury—Tension Pneumothorax	3.205320139	0	1	532	31,302
Primaryimpression_Vaginal Hemorrhage	3.205320139	0	1	532	31,302

## Discussion

The Naïve Bayes machine learning algorithm has substantially reduced the burden of identifying agricultural injury cases in PCRs by decreasing the number of records that need to be reviewed by visual inspection. Previous research showed that PCRs yield a higher proportion of occupational injury records than other types of existing administrative records, such as hospital data [24]. Therefore, speeding up the process of identifying cases without sacrificing accuracy is a significant advancement in the field of injury surveillance for agriculture.

By moving from simple keyword searches to employing the use of machine learning techniques, we have advanced much closer to achieving an operational, sustainable surveillance system using existing data. The healthcare system is already burdened and lacks additional time for reporting; therefore we embarked on this endeavor utilizing data that can be imperfect, understanding that formatted variables are often left blank or not required. The onus has been on us to enhance the ability to find cases in the vast number of existing records, instead of insisting that emergency services fill out yet another report.

We anticipate that PCRs will continue to be a stable data source for years to come. Advancements in electronic reporting have improved over the last decade with rural areas obtaining improved connectivity by way of broadband internet. Most PCR data are used for quality assurance and quality control for emergency medical services, but it is increasingly being seen as a viable research dataset [17, 25, 26]. In addition, state-based Emergency Medical Services Bureaus are interested in utilizing the results of research involving PCRs as a way to enhance EMS response.

It is possible to use a Naïve Bayes machine learning algorithm to identify agricultural injury records in Maine and New Hampshire. Utilizing two states and three years of data respectively, we examined how such a surveillance system performs over time, and how additional states may be added to the system in the future. In the case of cross-year and cross-state train/test splits, the necessary false positive rates were higher than with purely random splits, but still significantly better than chance (Table 4). The model performed best when all the variables were available, though it performed much better than chance when presented with 1) only the narrative or 2) only the categorical variables. This has major implications for expanding the surveillance system to new states, as the variables available through research data use agreements can vary by state. Results of cross-state train/test splits also suggest that while a model trained on one state does not generalize to another state perfectly, it may be an acceptable low-cost alternative to creating a state-specific training set. In addition, findings indicate that a model can be trained on earlier years and still generalize well to later years.

There is a slight decrease in the model’s performance when it’s applied to the newer years. Within the newer validation data, 30.5% of records were tagged for visual inspection (due to a posterior probability > 0.016) versus 15% in the training dataset. The variables which had the greatest discriminatory power included stemmed keywords which are quite unique to agriculture, for example hoov(e), silag(e), and grain_bin. While they showed up rarely in the PCR narratives, when they did they were a good indicator of an agricultural injury. Other variables that were strongly indicative of agricultural cases and appeared much more often include the keywords cow, hay, and pastur(e), as well as the incident location of Farm. The cost for maintaining this surveillance system can be reduced in two ways: by enhancing the accuracy of the machine learning algorithm and by altering protocols for visual inspection of records. Further review of the inter-rater reliability between coders will determine if we can reduce the time spent on visual inspection, without sacrificing significant errors in case classification.

Our ability to add states to the system and continue to review and code timely data will rest on continued refinement of the machine learning algorithm. To this end, next steps include the exploration of active machine learning. Part of this process is scrutinizing how much tagged data is necessary to get a new state off the ground, or to review how well the algorithm performs over time, understanding that databases and their data dictionaries evolve over time.

The descriptive epidemiology of the injury events identified will be the subject of a separate manuscript.

### Limitations

This surveillance method captures traumatic injuries for which EMS were involved, where the record contained a variable or keyword related to agriculture. Inherently, this leaves out injuries where medical treatment was sought without EMS involvement, such as those transported to the hospital in a private vehicle. This system is designed to capture ninety percent of true positive cases, knowing that some cases (10%) will be missed.

Since the model’s performance in later years does not exactly match that of the training years, further assessment is needed to understand if the full 90% of case-class 1, 2, and 3 are still captured in later years. In 2008–2010, the percent of records that made it to the model which were tagged & visually confirmed was 1.59% (15% * 10.6%). In 2011–2015, that was only 0.976% (30.5% * 3.2%). Assuming the base rate of true cases amongst records that make it to the model remains relatively constant over the nine year period, means that we are identifying a slightly smaller percent of true positives in 2011–2015 than in 2008–2010.

To refine the machine learning algorithm, a certain amount of tagged data is required, and the initial step of tagging the large corpus is quite time consuming. For the newer datasets to which the algorithm was applied, we cannot confirm that this dataset in fact contains ninety percent (90%) of all true cases. Understanding that would require visually inspecting a vast number of cases and requires further study. Choosing a lower required true positive rate will reduce the number of false positives that need to be reviewed, but will also increase the number of false negatives.

## Conclusions

This research adds substantial information to improving occupational injury surveillance using existing data sources. The application of the trained algorithm on newer data left less than two percent of records requiring visual inspection, making it a sustainable and cost-effective way to understand injury trends in agriculture. This system, along with companion surveillance methods such as those utilizing hospital, trauma and survey data, provides a broader picture of worker injury in agriculture. Continued investment in robust injury surveillance methodologies will benefit worker health and safety, by allowing occupational health and safety specialists to make informed decisions about hazards and evaluate the effect of injury prevention efforts over time.

## Data Availability

The data that support the findings of this study are available from the Maine and New Hampshire Bureau of Emergency Medical Services, respectively but restrictions apply to the availability of these data, which were used under license for the current study. Those interested in applying for these data may do so by contacting the respective EMS bureaus.

## Code availability

The algorithm developed by the authors is available by a written request to the corresponding author.
